# Acoustic Aposematism and Evasive Action in Select Chemically Defended Arctiine (Lepidoptera: Erebidae) Species: Nonchalant or Not?

**DOI:** 10.1371/journal.pone.0152981

**Published:** 2016-04-20

**Authors:** Nicolas J. Dowdy, William E. Conner

**Affiliations:** Department of Biology, Wake Forest University, Winston-Salem, North Carolina, United States of America; INRA-UPMC, FRANCE

## Abstract

Tiger moths (Erebidae: Arctiinae) have experienced intense selective pressure from echolocating, insectivorous bats for over 65 million years. One outcome has been the evolution of acoustic signals that advertise the presence of toxins sequestered from the moths’ larval host plants, *i*.*e*. acoustic aposematism. Little is known about the effectiveness of tiger moth anti-bat sounds in their natural environments. We used multiple infrared cameras to reconstruct bat-moth interactions in three-dimensional (3-D) space to examine how functional sound-producing organs called tymbals affect predation of two chemically defended tiger moth species: *Pygarctia roseicapitis* (Arctiini) and *Cisthene martini* (Lithosiini). *P*. *roseicapitis* and *C*. *martini* with intact tymbals were 1.8 and 1.6 times less likely to be captured by bats relative to those rendered silent. 3-D flight path and acoustic analyses indicated that bats actively avoided capturing sound-producing moths. Clicking behavior differed between the two tiger moth species, with *P*. *roseicapitis* responding in an earlier phase of bat attack. Evasive flight behavior in response to bat attacks was markedly different between the two tiger moth species. *P*. *roseicapitis* frequently paired evasive dives with aposematic sound production. *C*. *martini* were considerably more nonchalant and employed evasion in fewer interactions. Our results show that acoustic aposematism is effective at deterring bat predation in a natural context and that this strategy is likely to be the ancestral function of tymbal organs within the Arctiinae.

## Introduction

For over 65 million years night-flying moths have been locked in an evolutionary arms race with echolocating insectivorous bats [1]. Intense selection pressure on moths has led to the evolution of ultrasound-sensitive ears that allow early detection of the sonar signals of bats and aerobatic evasion [2]. As a second line of defense, tiger moths (Lepidoptera: Erebidae: Arctiinae) evolved sound-producing tymbal organs and the ability to answer bat echolocation cries with high-intensity, broadband clicks [3, 4]. Early observations and laboratory experiments have suggested that the tymbal sounds of some species can serve an aposematic function [5–12] with the moth sounds advertising the presence of noxious chemicals sequestered in the larval stages. It has also been suggested that aposematic clicks could serve a sonar jamming or weakly jamming function [9]. Previous studies with aposematic erebids were limited because they did not record bat and moth sounds, nor did they record video to analyze flight tracks quantitatively [6, 7]. We here use both sound and 3-D videography to address whether the tymbal sounds of *Pygarctia roseicapitis* (Tribe: Arctiini) and *Cisthene martini* (Tribe: Lithosiini) function as aposematic signals vis-à-vis bats (predominantly *Myotis* species) under field conditions in Southeastern Arizona. Among moths, there are two general types of anti-bat evasive flight maneuvers: “turn away” flight and “dives” toward the ground [13]. 3-D analyses similar to those used in this study have found comparable anti-bat maneuvers in insects including preying mantids, locusts, and other non-erebid moths [14, 15, 16]. Enacting these evasive maneuvers has been measured to increase the chances of escaping predation by 40–83% depending on the species [16]. The evasive behaviors of aposematic animals have never before been quantified. It is possible that aposematic animals will infrequently enact evasive maneuvers in response to the threat of predation, thereby exhibiting nonchalance. Alternatively, these organisms may escape predation by utilizing a diversified defensive portfolio that includes aposematic signaling in tandem with evasive maneuvering. We explore whether tiger moths produce warning sounds with or without evasive maneuvers, *i*.*e*. are tiger moths nonchalant or not? To our knowledge this is the first study of acoustic aposematism that allows for the 3-D reconstruction of the spatial interactions and coincidental recording of bat and moth sounds.

## Results

### Tymbal Sounds

**Fig 1:**
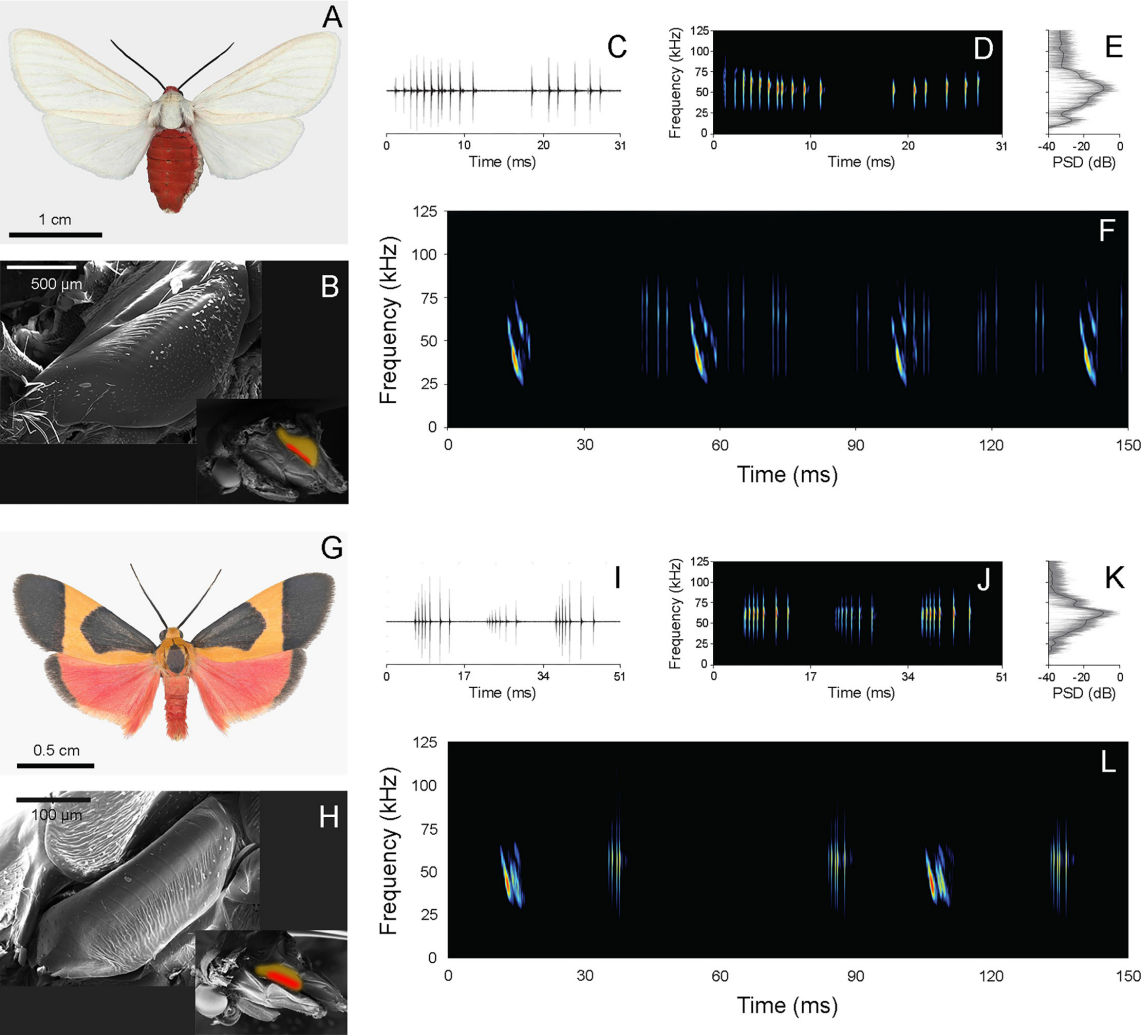
**Morphology and acoustic emissions of *Pygarctia roseicapitis* (A-F) and *Cisthene martini* (G-L).** The moths (**A, G**) and their corresponding tymbal organs (**B, H**), oscillogram (**C, I**), spectrogram (**D, J**), power spectral density plot (**E, K**), and the spectrogram of their response to simulated bat cries (**F, L**) are shown. Tymbal images are oriented with anterior on the left and ventral on the top with some scales removed. Insets show the relative position, orientation, and size of the tymbal (yellow) organ and microtymbals (red) on the thorax of each species. Insets are oriented with anterior on the left and dorsal on the top. Oscillogram, spectrogram, and power spectral density plots (**C-E, I-K**) show a single activation and relaxation (modulation cycle) of the tymbal organ. Moth responses to simulated bat cries (**F, L**) show each species’ earliest response and do not correspond to the same segment of time. Bat cries are brightest and sweep from higher to lower frequencies within a single call. Moth clicks are broadband and cluster in groups of clicks.

We found that *Pygarctia roseicapitis* and *Cisthene martini* activate their tymbal organs both in response to the pre-recorded echolocation attack sequences of the big brown bat, *Eptesicus fuscus* and during natural encounters with free-flying bats (mostly *Myotis* species). Sounds (**Fig 1**) are typical of arctiine erebids in that they are composed of a series of broadband clicks produced during flexion of the tymbal (active modulation half-cycle) and during relaxation of the tymbal (passive modulation half-cycle). Our measurements agree with those previously published [17]. *P*. *roseicapitis* has a peak frequency of 54.0±8.4 kHz, a maximum duty-cycle of 6.1%, and produces clicks with an intensity of 76.9±4.8 dB peSPL at 5 cm. *C*. *martini* has a peak frequency of 60.9±7.9 kHz, a maximum duty-cycle of 5.7%, and an intensity of 74.6±7.7 dB peSPL at 5 cm. Previous work contains additional acoustic measurements for each species (See Table 1 of [17]).

### Effects of Sound Production

**Fig 2:**
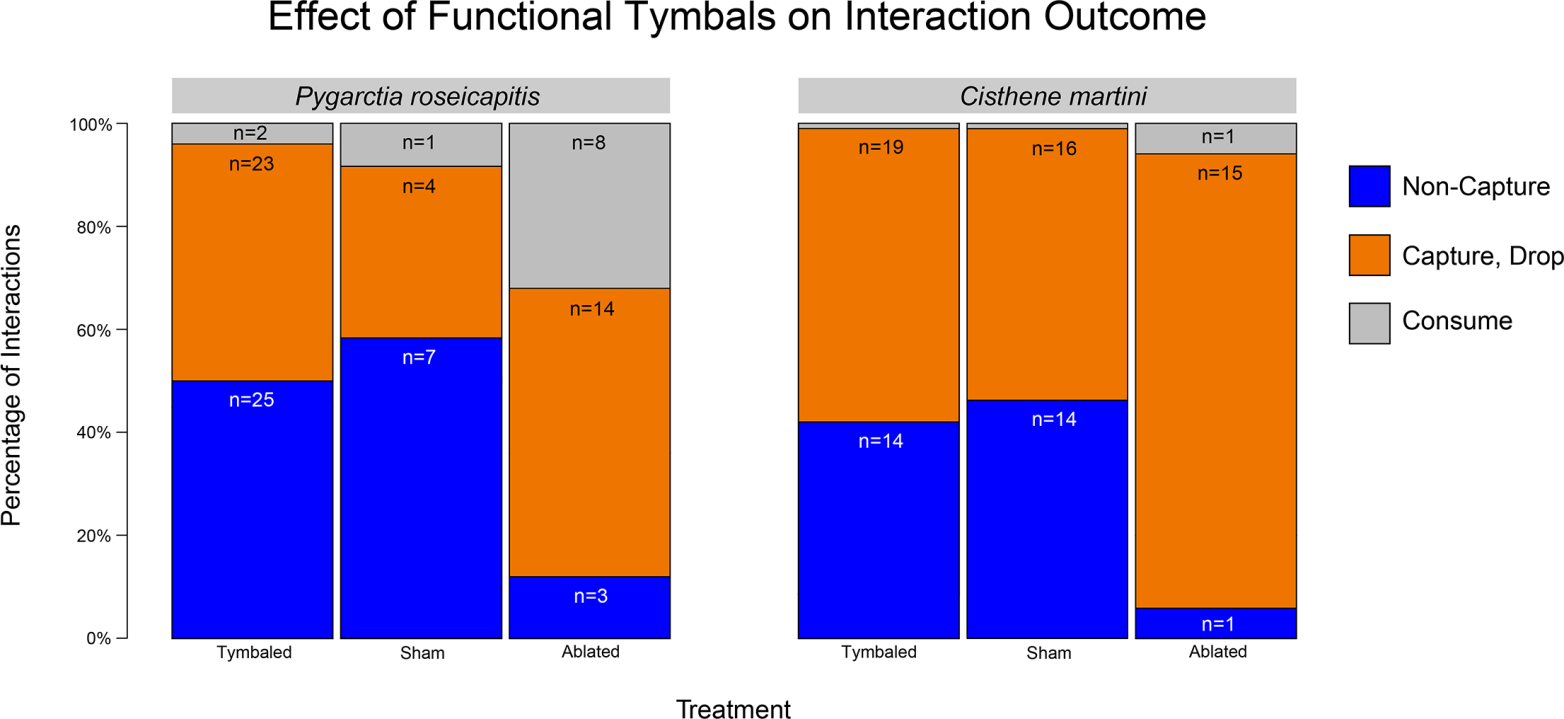
Effect of functional tymbals on the outcomes of bat-moth interactions for *Pygarctia roseicapitis* and *Cisthene martini*. The percentages of interactions for each possible outcome recorded for each treatment group. Numbers within each bar indicate the number of interactions observed for that treatment/outcome combination.

Functional tymbals had a significant influence on the outcome of bat-moth interactions involving either *P*. *roseicapitis* or *C*. *martini*. In both species, tymbaled control (T+ group) and sham operated moths (S group) had a lower relative risk of capture than their ablated (T- group) counterparts **(Fig 2)**. Statistical comparisons combine the “Capture, Drop” and “Consume” outcomes.

The relative risk of capture for tymbaled *P*. *roseicapitis* was 1.8 times less than their ablated counterparts (Fisher’s exact test: p = 0.01, odds ratio = 7.15, 95% C.I. = [1.8, 42.0]). Bats did not capture *P*. *roseicapitis* with intact tymbal organs in 50% of interactions (n = 25/50) while those with ablated tymbals were not captured in only 12% (n = 3/25). No significant difference in relative risk of capture was observed between the sham-operated controls (n = 5/12) and the tymbaled group (p = 1). The relative risk of capture between sham-operated and ablated individuals was significant (Fisher’s exact test: p = 0.02, odds ratio = 9.44, 95% CI = [1.5, 78.3]).

The relative risk of capture for tymbaled *C*. *martini* was 1.6 times less than their ablated counterparts (Fisher’s exact test: p = 0.03, odds ratio = 11.32, 95% CI = [1.4, 527.6]). Bats did not capture *C*. *martini* with intact tymbal organs in 42% of interactions (n = 14/33) while those with ablated tymbals were not captured in only 6% of trials (n = 1/17). No significant difference in relative risk of capture was observed between the sham-operated controls (n = 16/30) and the tymbaled group (p = 1). The relative risk of capture between sham-operated and ablated individuals was significant (Fisher’s exact test: p = 0.02, odds ratio = 13.35, 95% CI = [1.7, 626.0]).

Between *P*. *roseicapitis* and *C*. *martini* there was no difference in the relative risk of capture between tymbaled controls (p = 1), sham-operated controls (p = 1), and the ablated treatment groups (p = 1).

We examined the field recorded audio for 58 interactions which were reconstructed in 3-D and found that among interactions involving moths with intact tymbal organs (T+ and S treatments; n = 42) we were able to detect moth clicks 48% of the time (n = 20/42). We detected *P*. *roseicapitis* clicks in 50% of examined interactions (n = 14/28) and *C*. *martini* clicks in 43% (n = 6/14). This was not a significant difference in detectability between species (p = 1). We found that detecting the responses of these moths was challenging in a field setting and these percentages should be considered minimum estimates. We never detected moth clicks in interactions involving ablated moths of either species (n = 0/16).

### How did moths respond to bat attacks?

**Fig 3:**
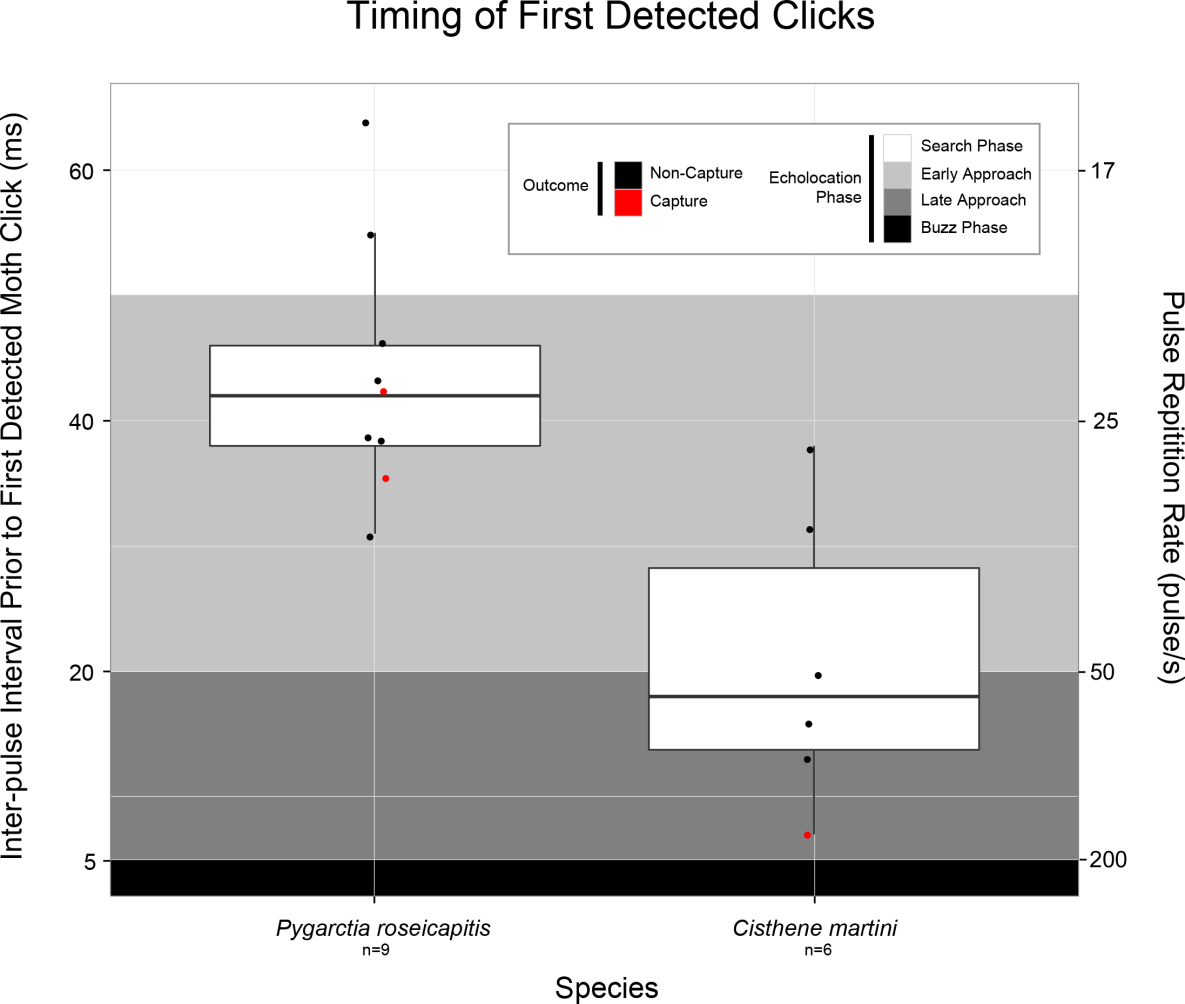
Inter-pulse interval (IPI) between the two bat calls immediately preceding the first detected moth clicks for “Tymbaled” (T+ and S groups), sound-producing *Pygarctia roseicapitis* and *Cisthene martini*. Box plot upper and lower hinges represent the 25^th^ and 75^th^ percentiles of their respective distributions. The 50^th^ percentile (median) is shown as a thicker black line between hinges. Tukey-style whiskers extend from each hinge to the most extreme value within 1.5*IQR (inter-quartile range). Actual data from which the box plots are constructed are displayed as points jittered along the midline of their respective box plot. Any data points beyond the whiskers are outliers. “Non-Capture” outcomes are colored black and “Capture” outcomes are colored red. Bat attack phases and their corresponding range of IPI’s are indicated as: Search Phase (white), Early Approach (light grey), Late Approach (dark grey), and Buzz (black). The right y-axis are the values of pulse repetition rate (pulse*sec^-1^) corresponding to the values of IPI.

To characterize how moths responded to bat cries under natural conditions we examined the inter-pulse interval (IPI) of bat cries immediately before the first detected moth clicks. The IPI is defined as the time elapsed between the onset of two sequential bat cries. IPI values for *Myotis spp*. in our recordings ranged from 5–120 ms. This analysis included only those interactions where moth clicks were detected before bat-moth minimum distance (n = 15/20) (**Fig 3**).

The mean IPI immediately before the first detected click was 44±3 ms for *P*. *roseicapitis* and 21±5 ms for *C*. *martini*. This difference was statistically significant (Welch t-test: p = 0.003, t = 3.91, df = 10, 95% CI = [10, 36]). This places the responses of *P*. *roseicapitis* in the early approach phase and the responses of *C*. *martini* later, near the beginning of the late approach phase of the bat attack. There was also a trend towards a higher likelihood of “Non-Capture” outcomes in interactions with clicks generated in earlier attack phases. Interactions with *P*. *roseicapitis* resulted in “Non-Capture” in 100% of trials when clicks were detected in search phase (n = 2/2), but only 72% of trials where clicks were detected in early approach phase (n = 5/7). Interactions with *C*. *martini* resulted in “Non-Capture” in 100% of trials where clicks were detected in early approach phase (n = 2/2), but only 75% of trials where clicks were detected in late approach phase (n = 3/4).

For each interaction where clicks were detected before bat-moth contact (n = 15/20), we calculated the bat-moth distance from the 3-D data at the time of the first detected click. The mean bat-moth distance at the first detected click was 151±49 cm for *P*. *roseicapitis* (n = 9/15) and 43±8 cm for *C*. *martini* (n = 6/15). The clicks of *P*. *roseicapitis’* were, on average, detected at a significantly larger bat-moth distance compared to *C*. *martini* (Welch’s t-test: p = 0.06, t = 2.14, df = 8, 95% CI = [-7, 222])).

Moth clicks rarely occurred in the theoretical 2 ms critical time window required to produce a sonar jamming effect [18, 19]. When clicking moths were avoided (n = 12/15), 86±3% of bat echoes were unaffected. When clicking moths were captured (n = 3/15), 87±6% of bat echoes were unaffected.

### How did bats respond to moth clicks?

**Fig 4:**
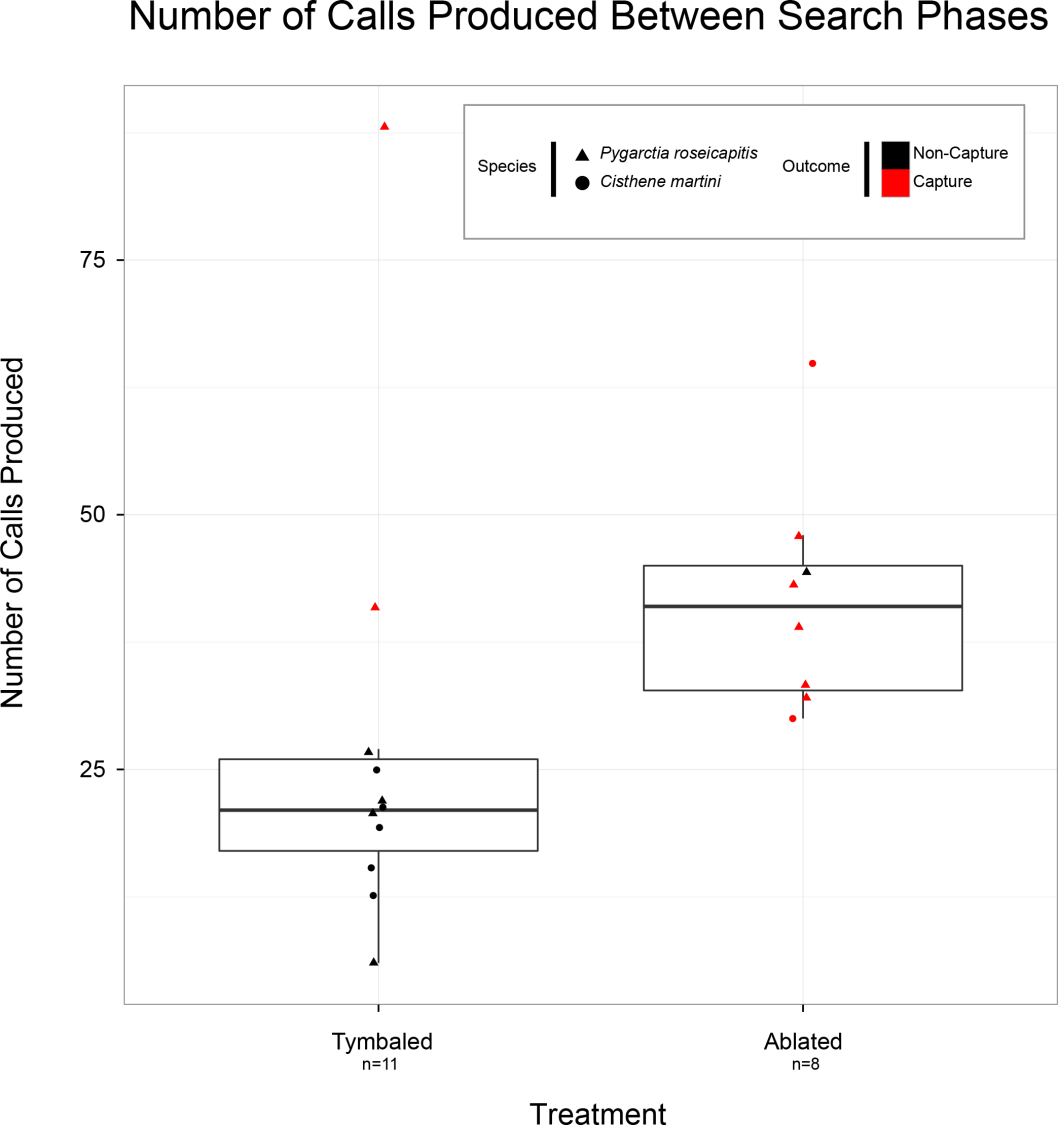
Number of echolocation calls bats produced between search phases for “Tymbaled” (T+ and S groups), sound-producing *Pygarctia roseicapitis* and *Cisthene martini*. Boxplot follows plotting conventions in Fig 3. For each interaction, moth species identity has been coded as shape and the outcome of each interaction is coded by color.

In order to assess how bats responded to moth clicks we compared bat echolocation behavior in a subset of interactions involving sound-producing tymbaled moths (T+ and S treatments) against ablated controls for *P*. *roseicapitis* and *C*. *martini* combined (n = 19). If a bat aborts an attack sequence and returns to search phase they will produce fewer echolocation calls compared to a completed attack sequence containing a terminal buzz. Therefore, we used the number of echolocation calls produced by bats in each interaction as a measure for whether bats aborted attacks in response to moth clicks. We began counting calls once the approach phase started (defined as <50 ms IPI) and stopped counting calls once the bats returned to search phase (>50 ms IPI) (**Fig 4**).

We found that bats produced a significantly lower number of calls when they were hunting moths that produced clicks (MWW test: W = 84, p = 0.01). Interactions with ablated moths averaged 42±4 calls per interaction (n = 8/19) while interactions with sound-producing moths only averaged 27±7 calls (n = 11/19). In this analysis, the only interactions in the T+ and S groups which resulted in “Capture, Drop” outcomes were outliers. The single ablated moth interaction which resulted in a “Non-Capture” was interestingly the only interaction to involve a lasiuriine bat (*Lasiurus sp*.). The bat appeared to have failed capturing the moth due to the moth’s evasive maneuvering (number of calls produced = 44).

### Palatability

The proportion of consumed moths in the ablated treatment groups are the best representations of palatability to a bat predator because they allow palatability to be separated from the confounding effects of sound. Both *P*. *roseicapitis* and *C*. *martini* appear to be highly unpalatable. When captured, silenced *P*. *roseicapitis* and *C*. *martini* were rejected in 64% (n = 14/22) and 94% (n = 15/16) of trials, respectively (Exact binomial test: p < 0.001, 95% CI = [0.44, 1.0]; p < 0.001, 95% CI = [0.74, 1.0], respectively). These values are similar to hand-feeding trials performed with these species using *E*. *fuscus* (Corcoran and Dowdy, unpublished data).

### 3-D Analysis of Bat-Moth Interactions

#### Bat flight behavior in response to sound

**Fig 5:**
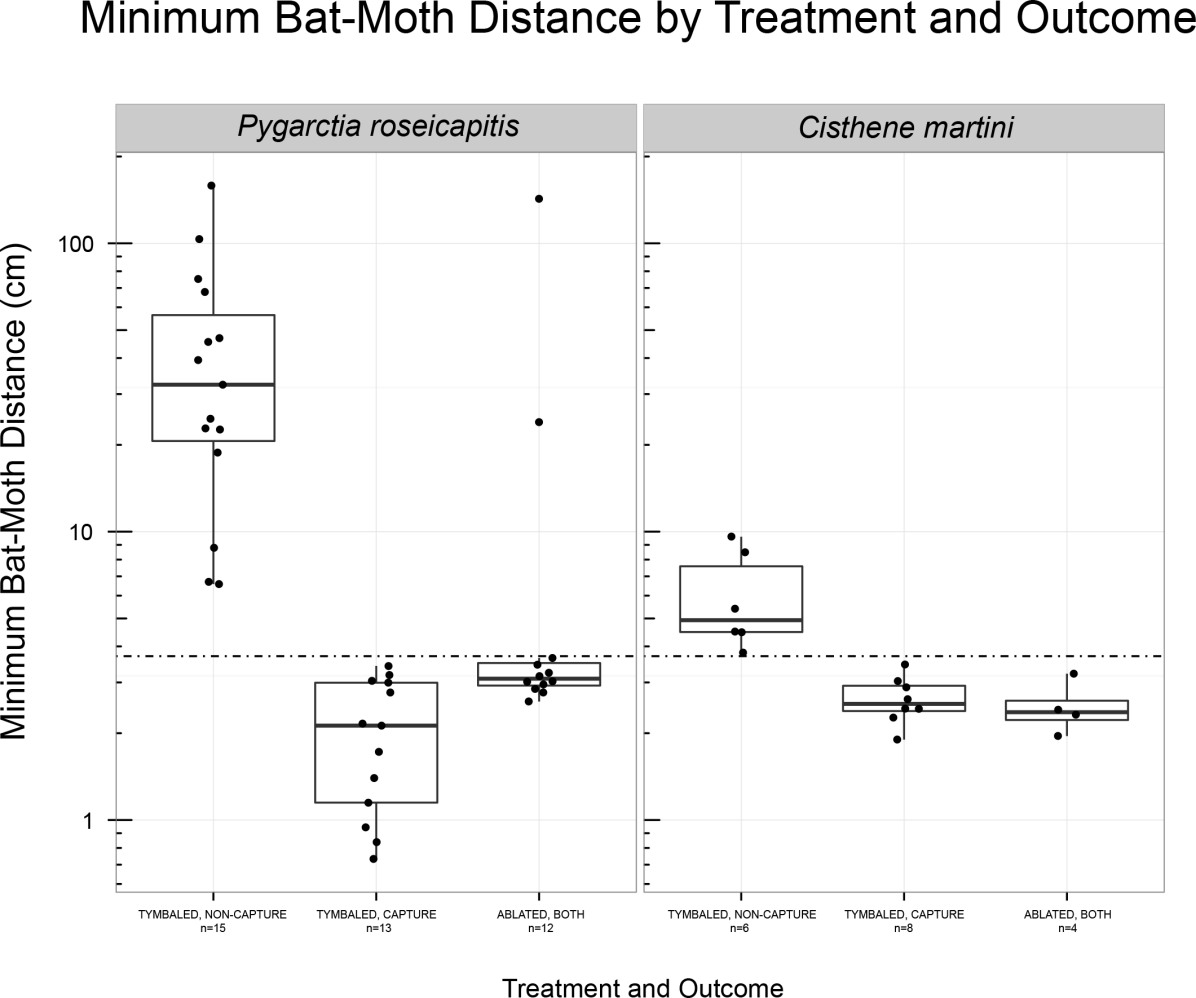
Minimum bat-moth distances (mBMD) between “Capture, Drop” and “Non-Captured” outcomes among “Tymbaled” (T+ and S groups) and “Ablated” (T-) moths for *Pygarctia roseicapitis* and *Cisthene martini*. Boxplot follows plotting conventions in Fig 3. Horizontal dot-dashed line demarcates 3.7 cm which was the most conservative of the smallest minimum bat-moth distances in which we could measure due to inherent limitations and error in the 3-D reconstruction process. Bat and moth should be considered to be occupying the same coordinates below this value. This plot displays the closest distance between bats and moths (T+ and S treatments) during each interaction. Interactions that resulted in “Capture, Drop” were all below 3.7 cm. All interactions that resulted in “Non-Capture” were above 3.7 cm.

The minimum bat-moth distance (mBMD) is a measure of the closest distance between the bat and moth during an interaction. We compared tymbaled (T+ and S) moths that were and were not captured to ablated (T-) moths to see how bat behavior differed (**Fig 5**).

Among all treatments, the mBMD of captured and non-captured moths of both *P*. *roseicapitis* and *C*. *martini* were significantly different (K-S test: p < 0.001, p < 0.001, respectively). When bats did not capture the moths their mBMD was large for *P*. *roseicapitis* ($\bar{x}$ = 33 cm, 95% CI = [7, 146]) and smaller for *C*. *martini* ($\bar{x}$ = 6±1 cm).

#### Moth flight behavior in response to bat predation

To determine whether *P*. *roseicapitis* or *C*. *martini* take evasive action in response to predator attacks or not, we quantified their evasive flight behaviors. We included trajectory data from 0–333 ms prior to the bat-moth minimum distance of each interaction. This time period was chosen because it encompasses the approach and buzz echolocation phases of a typical bat attack observed at our field site, during which moths are most likely to exhibit evasive behavior. This analysis includes only “Diving” behaviors as “Turn Away” flight was rarely observed.

**Fig 6:**
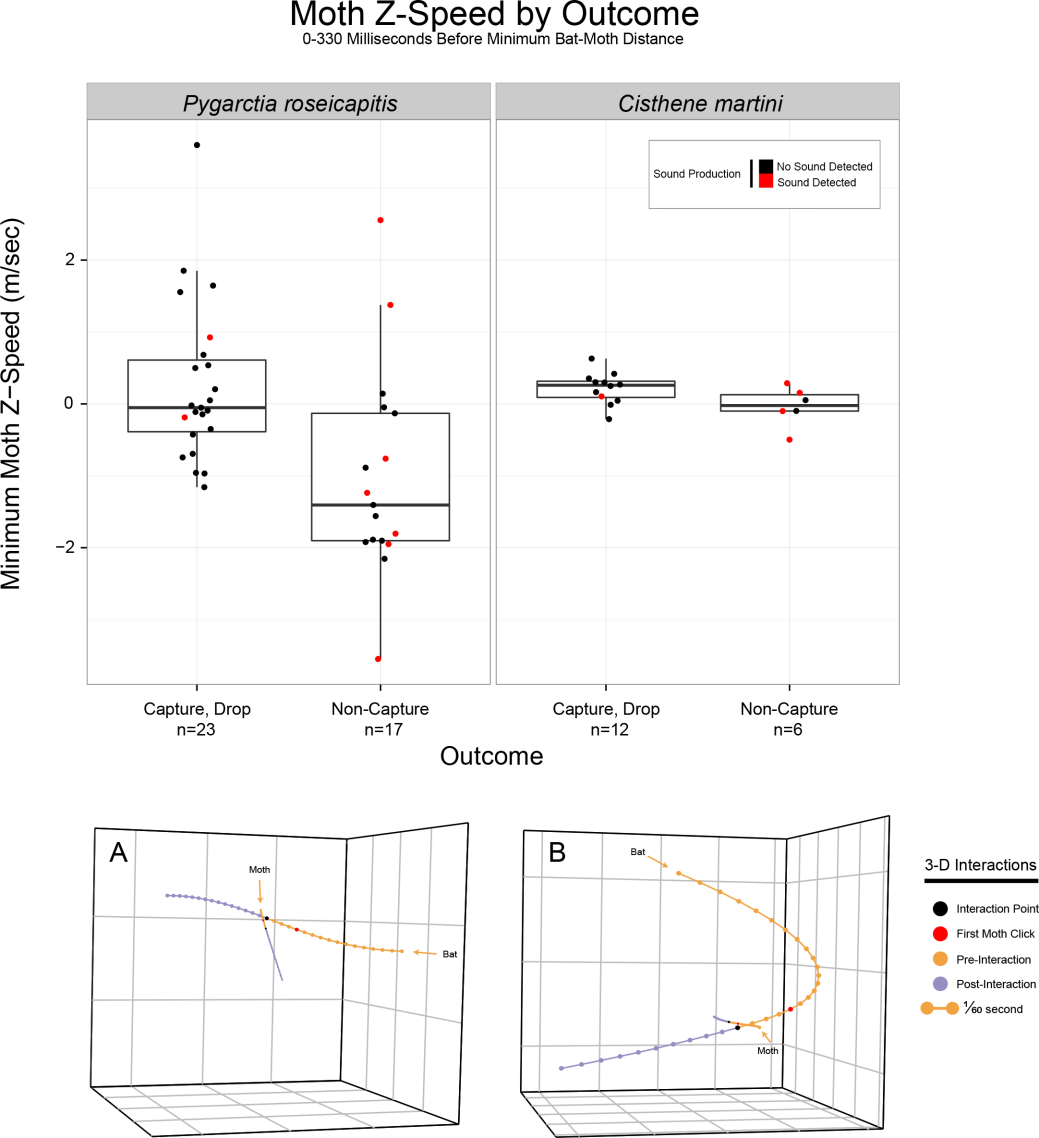
Moth z-speed between “Capture, Drop” and “Non-Captured” outcomes among tymbaled (T+ and S groups) moths for *Pygarctia roseicapitis* and *Cisthene martini*. Boxplot follows plotting conventions in Fig 3. The speed of the moths in the z-axis acts as a proxy for detecting diving evasive behavior. Positive values are upward flight, values near 0 m*sec^-1^ are level flight, and negative values are downward flight. Only *P*. *roseicapitis* which were not captured were significantly different from 0 m*sec^-1^, indicating that that species employed evasive dives. Neither outcome involving *C*. *martini* was significantly different from 0 m*sec^-1^, indicating that this species did not frequently employ evasive dives. 3-D perspective plots display representative flight path data. Bats are depicted as larger points and moths as smaller points. Starting points are indicated by an arrow. Time flows from Yellow (Pre-Interaction) > Black (Interaction) > Purple (Post-Interaction). Black points are the closest distance between bat and moth and red points indicate when the moth was first detected to click. **(A)** “Non-Captured” *P*. *roseicapitis* diving (negative moth z-speed) in response to a bat attack. **(B)** “Non-Captured” *C*. *martini* taking no evasive action (moth z-speed ≈ 0) in response to a bat attack. Neither bat turned away from the clicking moth nor did they enact typical prey capture behaviors.

The speed of the moths in the z-axis acts as a proxy for detecting diving behavior (**Fig 6**). Positive values are upward flight, values near 0 m*sec^-1^ are level flight, and negative values are downward flight. This calculation is similar to those employed in recent 3-D analyses of insect flight trajectories to quantitatively define diving behavior [15, 20]. A mean near 0 m*sec^-1^ would indicate nonchalant behavior whereas a significantly more negative mean would indicate a diversified defensive strategy that includes evasive dives.

We found that non-captured *P*. *roseicapitis* had a mean speed significantly more negative than 0 m*sec^-1^ ($\bar{x}$ = -1.01±0.35 m*sec^-1^). The mean z-speed of captured *P*. *roseicapitis* was not significantly different from 0 m*sec^-1^ ($\bar{x}$ = 0.19 m*sec^-1^, 95% CI = [-0.96, 1.83]). Both captured and non-captured *C*. *martini* had a mean that was not significantly different from 0 m*sec^-1^ ($\bar{x}$ = 0.21±0.06 m*sec^-1^, $\bar{x}$ = -0.03±0.11 m*sec^-1^; respectively).

**Fig 7:**
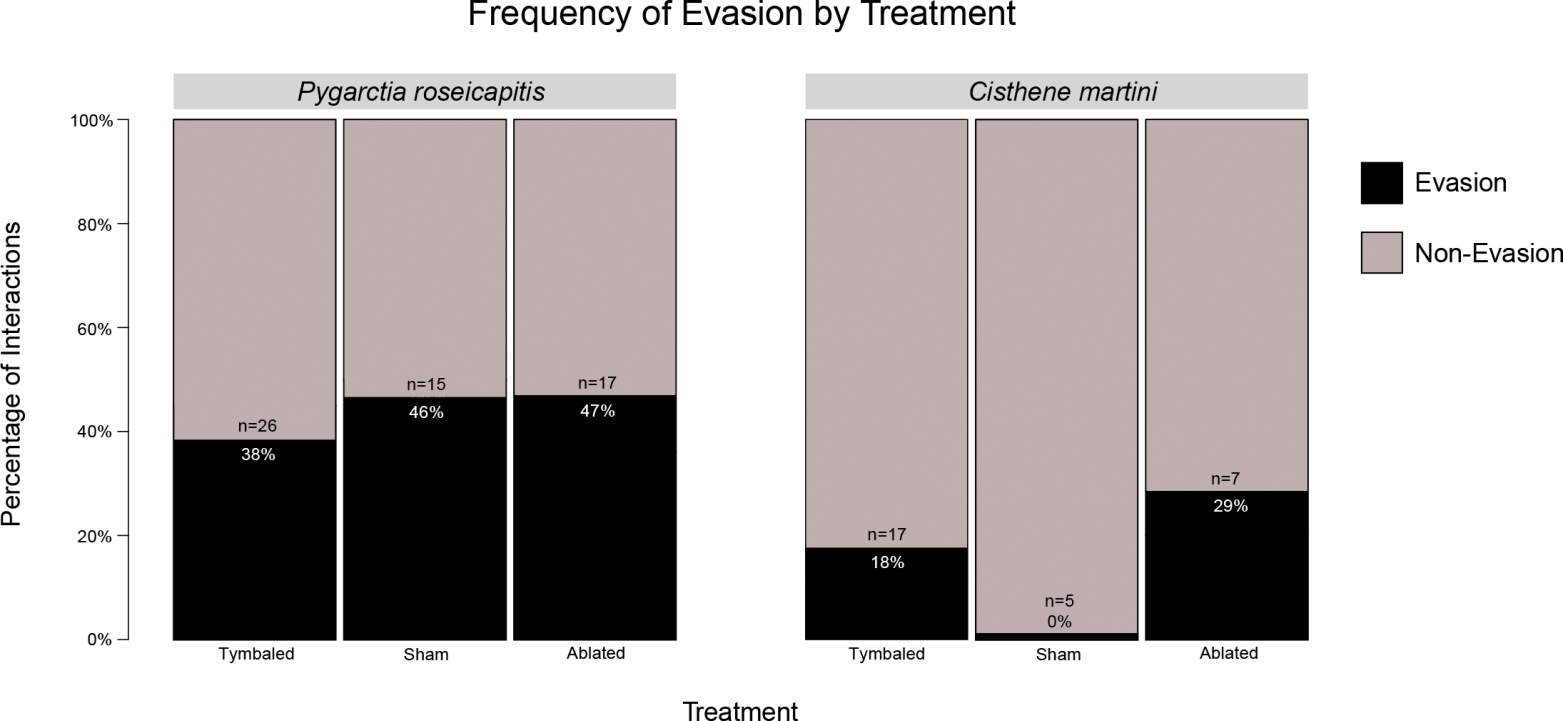
Percentage of interactions hand-scored as “Evasion” by treatment for *P*. *roseicapitis* and *C*. *martini*. Numbers within each bar indicate the number of interactions observed for that treatment group and percentages indicate the percent of those observations that were scored as “Evasion”.

To obtain a better estimate of the frequency of evasion among species and treatments we qualitatively examined the evasive behaviors exhibited by moths in the 58 3-D interactions as well as 36 additional interactions that were not included in our 3-D analysis (n = 94). These interactions were chosen because they had sufficient footage before and after each interaction to accurately identify the presence or absence of evasive maneuvers, only included interactions involving a single moth and a single bat, and only included interactions where a moth’s evasive flight could be easily classified. Based on visual classification these interactions were scored as either “Turn Away”, “Dives”, or “No Evasion” as defined in classic studies of moth evasive flight [13]. Among these 94 interactions, only 7% (n = 7/94) exhibited “Turn Away” behavior. For this reason we restricted our analysis to include only moths exhibiting “Dives” (renamed “Evasion”) or “No Evasion” (n = 87) (**Fig 7**).

The treatment groups (T+, T-, S) of *P*. *roseicapitis* and *C*. *martini* exhibited the same number of evasive dives (Fisher’s exact test: p = 0.84; p = 0.66, respectively). To compare the rate of evasion between species, we have pooled the treatment data for each species because treatment did not affect whether evasion was performed. *P*. *roseicapitis* employed evasion 2.5 times more often compared to *C*. *martini* (43% versus 17%; Fisher’s exact test: p = 0.02, odds ratio = 3.58, 95% CI = [1.12, 13.74]).

## Discussion

This is the first study of aposematism within the bat-moth arms race in which both the bat and moth were tracked in 3-D space and acoustically recorded in nature. Our results are consistent with the idea that tymbal sounds produced by some tiger moths can act as acoustic warnings of underlying chemical defenses, *i*.*e*. aposematic signals. Both *P*. *roseicapitis* and *C*. *martini* respond to bat predation by making similar tymbal sounds under laboratory and field conditions. These sounds proved effective in reducing their predation by local free-flying bats (predominantly *Myotis* species). With tymbals intact, *C*. *martini* were 1.6 times less likely to be captured by a bat and *P*. *roseicapitis* were 1.8 times less likely. Bats generally kept their distance from sound-producing moths, did not enact prey capture behaviors, and increased the inter-pulse intervals of their echolocation calls after failing to produce buzz phase calls, which all suggest that they actively aborted their attacks. When moths were captured by bats they were often rejected unharmed, indicating that both moth species studied are rendered relatively unpalatable, likely by a short-range chemical-based secondary defense mediated by predator olfaction, gustation, or a combination of the two. Our field results are consistent with earlier laboratory findings that bats can associate tymbal sounds with noxious moths and thereafter avoid them [10–12].

*P*. *roseicapitis* is a member of a clade of aposematic species that includes *Cycnia tenera* [21], a species that has been shown unequivocally to be aposematic in laboratory experiments. Both species feed on cardiac glycoside-containing plants, likely sequester similar compounds, and are unpalatable to bat predators [22–24]. The peak frequency, maximum duty-cycle, and intensity of the sounds produced by *P*. *roseicapitis* and *C*. *tenera* are similar (See Table 1 of [17]). Thus it appears that they both use aposematic strategies to deal with insectivorous bats. Likewise, *C*. *martini* and *Hypoprepia fucosa* (from [6, 7]) are members of a clade of erebids that are unpalatable based on their shared sequestration of secondary chemicals from lichens [25]. As above, the tymbal sounds of these two species are similar (See Table 1 of [17] and Table 2 of [26]) and appear to function as warning sounds.

It has been suggested that aposematic clicks could serve a jamming or weakly jamming function [9]. Most research suggests that jamming occurs via ranging interference [18, 19, 27, 28]. The duty-cycles of the sounds produced by *P*. *roseicapitis* and *C*. *martini* are much lower than those of *Bertholdia trigona* [17], the only proven sonar jammer (~6% versus 44%) and because of this we argue that it is unlikely that these sounds produce a strong jamming effect. In interactions with *P*. *roseicapitis* and *C*. *martini* over 85% of bat echoes were unaffected by moth clicks, suggesting a weak jamming effect at best. This further supports an aposematic function for the sounds produced by *P*. *roseicapitis* and *C*. *martini*.

*P*. *roseicapitis* and *C*. *martini* produced sounds in response to inter-pulse intervals associated with the approach phase of bat echolocation. Studies with the dogbane tiger moth, *C*. *tenera* showed they responded most often to a pulse repetition rate of 30–50 calls*sec^-1^ which is equivalent to an inter-pulse interval of 20–33 ms [29]. Our results include responses within the range reported for *C*. *tenera*. However, the mean IPI that *P*. *roseicapitis* responded to was much longer while *C*. *martini* was near the lower limit of this range. A more recent study that examined the timing of responses within a diverse group of neotropical tiger moths showed that species can vary drastically in the timing of their responses [30]. The difference in timing of clicks between *P*. *roseicapitis* and *C*. *martini* is therefore not unexpected and both species’ first responses fall within the range reported for other tiger moths. It is unknown what factors might contribute to variation in the timing of acoustic responses and without a well-supported phylogeny analyzing this data while taking into account shared ancestry is impossible.

The bat-moth distance when *P*. *roseicapitis* and *C*. *martini* clicks were first detected were somewhat smaller than those reported for *B*. *trigona* (See Fig 6A of [20]). These species are much quieter and produce fewer clicks per unit time compared to *B*. *trigona* so it is possible that this could be explained by a bias towards later detection times in this study. In interactions where moths were detected to click the earliest, we did not observe major changes in bat trajectories or “turn aways” as has been described in other 3-D field studies in response to sonar jamming signals [20]. Instead, the bats in this study typically made relatively minor adjustments to their trajectories, but did not make capture attempts. This behavior has been recorded in lab conditions with *C*. *tenera* against the bat *E*. *fuscus* (Hristov and Conner, unpublished). *P*. *roseicapitis* produced clicks farther away and ended interactions farther from bats than *C*. *martini*. It is possible that this difference in minimum bat-moth distance could be attributed to *P*. *roseicapitis* clicking earlier, the greater use of evasion by *P*. *roseicapitis*, or a combination of the two.

We were surprised by how often both species were captured by the bats and then rejected compared to previous laboratory and field data where most were not captured [6, 7, 10, 11]. There are several possible explanations. Despite the fact that most of the moths were rejected by bats, the level of chemical defenses may not be high or they may be variable among individuals, *i*.*e*. automimicry. Another possibility is that the area, which is particularly speciose in moth fauna, may carry a high load of Batesian mimics. Either scenario may make a sample-and-reject strategy viable [31]. It is also possible that the bat population was dominated by inexperienced young of the year. These animals undoubtedly require time to learn the aposematic signal and to associate it with noxious prey. The bat-moth season in the Chiricahua Mountains is tied to the local monsoons and is particularly compressed in time (~4–6 weeks total per year). This may also be a contributing factor in that competition for food at this time of the year is particularly intense. This phenomenon could also be partly due to tymbaled moths failing to respond to bat echolocation for unknown reasons. We currently do not have sufficient data to test these possibilities.

It is possible that *C*. *martini* and *P*. *roseicapitis* are part of a larger acoustic Batesian, quasi-Batesian, and/or Müllerian mimicry ring(s) in Southeastern Arizona. We are in the process of collecting data on additional moth species in the area. The target taxa include *Carales arizonensis*, *Pygarctia murina*, and *Ctenucha venosa*.

In the only other field studies of acoustic aposematism, the moths, *H*. *fucosa* (Lithosiini) and *Halysidota tessellaris* (Arctiini) were said to fly straight with no obvious evasive maneuvers [6, 7]. In contrast, the responses of *P*. *roseicapitis* and *C*. *martini* to bat attacks are more varied and include aposematic signaling and, in some cases, evasive maneuvers like dives. The 3-D flight tracks and behavioral scoring show that nearly half of *P*. *roseicapitis* produce aposematic clicks in tandem with evasive dives. In comparison, *C*. *martini* can be considered nonchalant, diving in only 17% of interactions. This suggests that, with respect to evasive responses, species can lie in different places along a nonchalance continuum.

In those early field studies of aposematic erebids neither quantitative measurements of bat-moth flight trajectories nor the acoustic responses of moths to foraging bats were included. By recording and filming interactions between aposematic tiger moths and bats under natural conditions we have presented the first quantitative data detailing (1) how sound production influences the outcome of these interactions, (2) how these moths and bats alter their flight behaviors during these predation events, (3) how these moths time their acoustic signals, and (4) how bats change their echolocation in response. These results highlight the strengths of a quantitative, comparative approach in understanding the diversity of strategies within the bat-moth arms race. All aposematic tiger moths do not respond to bat predation the same.

Based on the most recent phylogenetic analysis of the subfamily Arctiinae (Family: Erebidae), *P*. *roseicapitis* and *C*. *martini* are members of two separate, monophyletic tribes (Arctiini and Lithosiini, respectively) [21]. The primary difference between these tribes is their larval feeding behavior. Arctiini feed on a variety of plants including those containing pyrrolizidine alkaloids and cardenolides [21] whereas members of Lithosiini feed as larvae on lichens and are known to sequester polyphenolic defenses, likely from the algal symbiont in the lichen [25]. Phylogenetically, the Lithosiini are positioned basal to the Arctiini. Our data suggest that acoustic aposematism may be a synapomorphic character for all members of the subfamily Arctiinae. This hypothesis should be tested with rigorous phylogenetic methods.

To fully understand the evolutionary history of the bat-moth arms race we need to examine the variety of anti-bat defenses deployed by arctiines on a larger scale and within a complete phylogenetic framework. This would add much needed resolution to the picture of how this predator-prey system has come to exist in its present form.

## Methods and Materials

### Ethics Statement

No vertebrates (bats) were captured or handled during these experiments. All data involves free-flying bats in their natural habitats. No state or federal permits were required to conduct this work. The methods of this study were approved by the Wake Forest University Institutional Animal Care and Use Committee (protocol #A12-048). This work was performed with permission on private property.

### Field Site

Field experiments were conducted at the Southwestern Research Station (SWRS) operated by the American Museum of Natural History. SWRS is located in Cochise County approximately 7 km southwest of Portal, Arizona, United States. The GPS coordinates of the field site are: 31°53’00.30” N 109°12’27.20” W; elevation: 1,650 m. This site was chosen for its high diversity of both bats and moths. The field trials were performed between July 18^th^ and August 10^th^ during 2011, 2012, and 2013.

### Moth Collection and Manipulation

Moths were collected on station grounds from sheets illuminated with 15 Watt ultraviolet “quantum” lights (Leptraps.com; F15T8QBL). Moths identified as either *Pygarctia roseicapitis* or *Cisthene martini* were stored individually for up to 24 hours in 30mL plastic containers at ambient temperatures. These species were targeted because they have previously been shown to produce sound in response to bat echolocation [17] and are thought to sequester defensive compounds from their larval hosts. Larval *P*. *roseicapitis* are known to feed on toxic *Euphorbia* species [23], and at SWRS we found them feeding on Desert Milkweed (*Asclepias angustifolia*). They can be reared on other cardiac glycoside-containing plants including *Apocynum cannibinum* (Dowdy, pers. obs.). Species of *Cisthene* and other Lithosiines have been reared on lichens which can contain polyphenolic compounds and those compounds have been found in the tissues of adults [25, 32, 33]. *P*. *roseicapitis* (Arctiini) has a forewing length of 1.4–1.7 cm and both fore- and hindwings are pearly white with a contrasting red head and abdomen. *C*. *martini* (Lithosiini) has a forewing length of 0.9–1.1 cm with orange and black coloration on the forewings, red coloration on the hindwings, and a red or orange abdomen **(Fig 1).**

Individuals were randomly placed into one of three treatment groups: Tymbals Intact (T+), Tymbals Removed (T-), and Sham Control (S). Moths from all three treatments were placed in individual vials and chilled for 5 minutes in an ice bath prior to surgery. The T+ group was removed from the ice bath and no further manipulations were performed. In the T- group tymbal organs were ablated with curved forceps by removing the cuticular surface of the organ. The space below the tymbal organ’s surface is a small, air-filled chamber, so this ablation did not cause any discernable injury or loss of haemolymph. Loss of tymbal function for this group was verified by manipulating the individual while monitoring for sound production with an ultrasonic detector (Pettersson Model D-100). In the S group the scales surrounding the areas of the tymbal organs were removed using curved forceps to simulate experimental manipulation without removal of the sound-producing organs. Individuals in all treatment groups were then assigned random ID numbers as designators such that their group assignment was not known to experimenters during field releases and data analysis. We cross-referenced ID numbers after data analysis was complete to match data with their respective treatment and species identities.

### Outdoor Flight Arena

Two ultraviolet lights were placed approximately 5 m off the ground and set 4 m apart in the center of an open grassy field. This area is approximately 600 m^2^ and surrounded primarily by Arizona sycamore (*Platanus wrightii*), scrub oak (*Quercus turbinella*), and alligator juniper (*Juniperus deppeana*). The ultraviolet lights served to increase general insect abundance at the field release site as well as to provide low-intensity ambient light to the area. The insects drew in free-flying bats which began to forage reliably in this outdoor flight arena. Moths included in this study were released at this site after treatment, one at a time, starting after sunset (21:00) for six hours (03:00) or until we ran out of moths to run in trials. The majority of releases involved the moths taking off shortly after being released from their containers. In a few cases we released the moths by tossing them up in the air. In these cases, we did not collect data for 5–10 seconds or until it was clear the moth was flying under its own power and would be able to react normally to any bat attacks. This was confirmed using the video recordings. We recovered any experimental moths that were not captured or that did not fly away in order to keep the number of experimental moths flying at one time to a minimum. There was almost always only one experimental moth flying at a time.

### Audio Recording and Analysis

Audio of the bat-moth interactions was recorded using three Avisoft Bioacoustics CM16/CMPA ultrasonic microphones (Berlin, Germany) with an Avisoft Ultrasound Gate 416H recording interface. Two microphones were placed approximately 1.5 m high and 4 m apart, near the edges of the recording volume, pointed up towards the interaction space. These stationary microphones aided in registering echolocation calls to bats filmed within the flight arena. The third microphone was mounted on a pole wielded by a central observer and maximized the likelihood of detecting moth sounds by minimizing the distance between the microphone and the moth. The pole was held approximately 1–3 m from the moth during field releases. All recordings were analyzed in Avisoft SASLab Pro v5.2.

Inter-pulse intervals (IPI) of bat calls for each interaction were determined by calculating the time elapsed between the two bat calls immediately preceding the first detected moth clicks. We have defined our bat attack phases in terms of IPI as: <5–7 ms (buzz), 8–20 ms (late approach), 21–49 ms (early approach), >50 ms (search). To convert IPI to pulse repetition rate we calculated and reported the number of calls (pulses) that would occur in 1 second at a given IPI. The time of the first detected moth click was cross-referenced with our 3-D flight path data to determine the bat-moth distance when the moth first clicked.

To determine whether moth clicks had the potential to jam bat echolocation we measured the frequency of their occurrence within the 2 ms critical window of each bat echo for the interactions reconstructed in 3-D [18, 19]. We used only echoes of bat calls that were produced after the search phase ended (<50 ms IPI) and before the next search phase began (>50 ms IPI) for each interaction. This allows for a standardized comparison to be made between interactions which vary in the number of bat calls they contain. For each bat call we used the middle of the call to represent the time point at which the call was emitted. This time point was cross-referenced with our 3-D flight path data to determine the bat-moth distance at that time. We then calculated when the echo of that call would return to the bat by assuming a speed of sound of 343 m*sec^-1^ and a travel distance of twice the bat-moth distance (time to travel to the moth and back). The critical window was calculated to be the echo’s return time point ±2 ms. We then calculated when the moth clicks would arrive at the bat. If any of the moth clicks occurred within the critical window we scored this as having the potential to jam that bat echo. To determine the number of unaffected bat echoes in each interaction we took the number of critical windows which were not overlapped by moth clicks and divided by the total number of bat echoes used in the critical window assessment.

### Video Recording

Videos of the interactions were recorded using three Basler AG Scout infrared cameras (Model scA640-120gc; Ahrensburg, Germany) at 60 frames*sec^-1^ with 640x480 resolution. The cameras were synchronized with the audio recordings using custom hardware (Innovision Systems, Columbiaville, MI, USA). Video was acquired with MaxTraq2D software (Innovision Systems) and two Intel PRO/1000 PT Dual Server Adapters (Intel, Model: EXPI9402PT) installed in a PC running Windows 7. Six Wildlife Engineering IR-Lamp6 lights (Tucson, AZ, USA), two Bosch UFLED20-8BD illuminators (Farmington Hills, MI, USA) and two Raytec Raymax 200 platinum illuminators (Ashington, UK) provided infrared illumination in the flight arena.

### 3-D Reconstruction

The relative orientation method was used to calibrate the cameras to perform three-dimensional (3-D) reconstruction of bat and moth trajectories [34]. These methods were applied in MaxTraq3D software (Dynamic Wand Method; Innovision Systems). Two spherical, infrared-reflecting markers were fixed a known distance apart on a T-shaped calibration wand. The calibration wand was rotated and translated throughout a subset of the flight arena’s volume, recorded, and digitized. An L-shaped calibration frame was also constructed to set the origin of the 3-D coordinate system. The calibration frame has four points set at a known distance from each other and is held motionless in one location within the recording volume during calibration. This configuration allowed for a calibration volume of approximately 90 m^3^ (4 m x 5 m x 4.5 m) with a maximum spatial error of 3.7 cm.

A visual observer tracked each released moth while that moth flew in the calibrated volume of the flight arena. An interaction was recorded if the visual observer within the calibrated volume (<1–2 m from moth) judged that a bat was flying near to the moth of interest and, in some cases, heard the bat’s terminal buzz calls. Further screening was performed on recorded interactions after filming to ensure that bats were flying towards released moths rather than flying past them. We found that most bats that were not interested in the released moth were either clearly interested in a different insect within the flight arena or flew much higher in an airspace that was not included in our recorded calibrated volume. We included only interactions involving one bat and one moth in our data analysis.

We filmed 167 bat-moth interactions with easily discernable outcomes. For 3-D analyses we used a subset of 58 interactions with the highest quality and lowest spatial errors. These bat-moth flight trajectories were digitized in MaxTraq2D. The locations of individuals were represented as single point centroids determined by a center of mass calculation. The digitized frames representing bat and moth locations through time were imported into MaxTraq3D for further conversion into 3-D (x, y, z) coordinates readable by MATLAB (Natick, MA, USA). A custom MATLAB script (BATracker; coded by Brad Chadwell) and a smoothing spline function (MATLAB spaps routine) were used to generate and smooth each flight path. Smoothed flight paths were then used to estimate flight parameters.

### Behavioral Scoring

Experimental moths were individually selected at random from the pool of available individuals. Moths were pre-warmed with a heat lamp to ~27°C to insure maximum flight performance and released from the center of the flight arena and tracked visually and recorded (video and audio).

**Fig 8:**
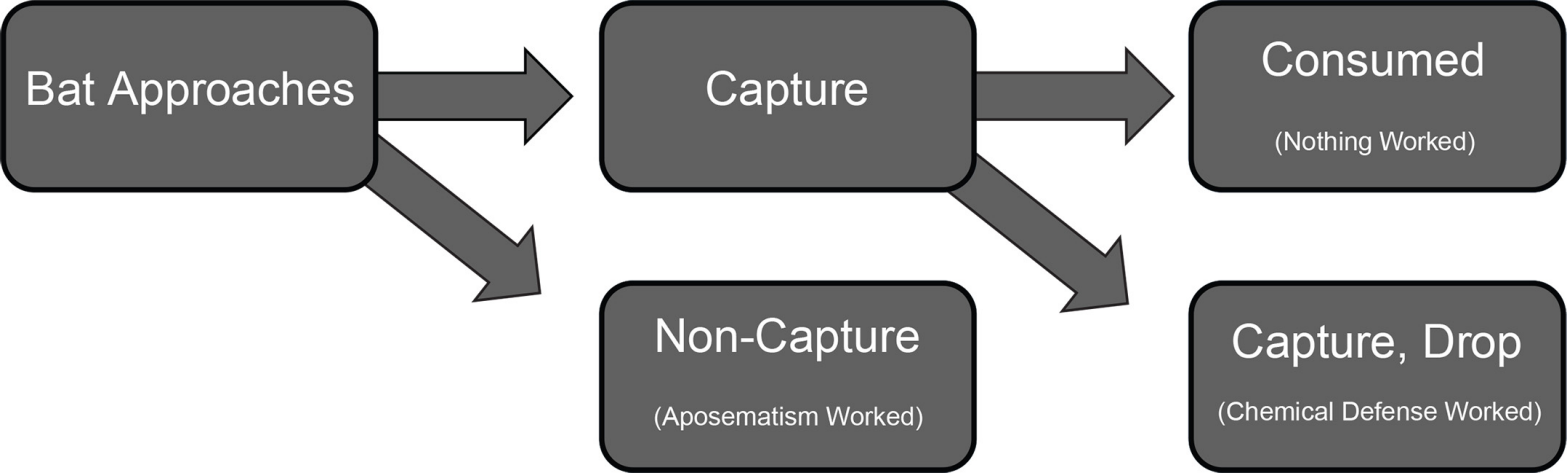
Ethogram showing the progression of bat attacks and possible outcomes of bat-moth interactions. A bat approaches a moth and can either capture or not capture that moth. If the moth is not captured that encounter has ended and the moth has survived. If the bat has captured a moth it can then either drop it or consume it. In this study’s context, if a sound-producing moth is not captured it is evidence that aposematism was effective in deterring the bat attack. If a captured moth is rejected by being dropped they typically survive and this is evidence that defensive chemistry was effective in deterring consumption.

Bat-moth interaction outcomes were separated into 3 categories (**Fig 8**). (1) Non-Capture: The bat turned toward the moth and closed in distance, but did not make contact with the moth. (2) Capture, Drop: The bat captured the moth in the tail or wing membrane and then released it. (3) Consume: The bat captured the moth in the tail or wing membrane and it was not released; assumed to have been consumed.

### Bat Species Identification

Automated acoustic species recognition software was utilized to identify the bat species foraging during data collection (Kaleidoscope Pro 2.0, Wildlife Acoustics, Inc., Concord, MA).The species classifiers for the Arizona region and the “+1 More Accurate” setting was used during classification. In addition, we verified these identifications by hand using call structure and frequency content. Of the 167 interactions in this study, 85% were determined to belong to the genus *Myotis*, 3% were lasiurine, 1% were *Eptesicus fuscus*, and 10% were unable to be confidently classified. The 58 digitized interactions were also predominantly (>85%) comprised of bats in the genus *Myotis*. Nearly all bats included in the analysis of the timing of moth clicks (**Fig 3**) and the number of bat echolocation calls produced between search phases (**Fig 4**) were verified to include *Myotis* exclusively. The only exception was a single data point from an ablated moth which was determined to involve a lasiurine.

### Lab Recording of Moth Sounds

Freshly captured moths were held by the wings folded above the thorax using a hemostat. All recordings were made in a darkened room. An Avisoft Bioacoustics USGH digital recording unit was connected to a single Avisoft CM16/CMPA ultrasonic microphone (± 3 dB from 15–140 kHz) and set to record at a sampling rate of 250 kHz. The microphone was placed perpendicular to the midline of the moth body, 10 cm from the thorax of the individual (where sound-producing organs are located). An AT 100 ultrasonic speaker (Binary Acoustic Technology) was placed 10 cm from the posterior end of the moth thorax (where tympanal hearing organs are located), parallel to the midline of the body. Moths were stimulated to produce sound by playing a pre-recorded echolocation attack sequence from the sympatric insectivorous bat, *E*. *fuscus*. Search, approach, and buzz phases of bat echolocation were all present and spanned a pulse interval of 115 ms in search phase to 6 ms in the buzz phase. Echolocation intensity reached and then sustained a peak equivalent Sound Pressure Level of 100 dB at 10 cm in the approach phase. For more details see previously reported methods [30]. Stimuli were repeated seven times per individual with approximately 4–5 seconds of silence between trials.

### Statistics

Statistical analyses of observation data as well as 3-D data were performed in R version 3.2.0 [35].

Means are reported with the standard errors on the mean. If distributions violated the assumption of normality the data were log_10_ transformed in order to fit a normal distribution as confirmed by the Shapiro-Wilk test and 95% confidence intervals are reported instead of standard errors on the mean. Fisher's exact test was used to test for independence between the three nominal treatment variables (T+, T-, S) and two nominal outcomes (“Non-Capture”, “Capture, Drop” combined with”Consume”). Fisher’s exact test was also used to test for differences in the frequency of evasion among treatments within species and between the two species.

Palatability was assessed using the Exact Binomial Test. Rejections by bats were coded as “successful” trials, the hypothesized null probability of rejection was 0% (perfectly palatable), and the alternative hypothesis was that the true probability of rejection was greater than 0%. Welch’s t-test was used to test for differences in the mean inter-pulse interval as well as the mean minimum bat-moth distance between species. The nonparametric Mann-Whitney-Wilcoxon test (MWW test) was used to test for a significant difference in the number of calls produce by bats attacking sound-producing tymbaled versus ablated moths. When testing for differences in some 3-D trajectory analyses the assumption of normality was violated. In these cases we used the non-parametric Kolmogorov-Smirnov test, referred to as the K-S test in this text.

Tests for unequal variances were performed using Levene’s Test implemented in the lawstat package for R after removal of outliers [36]. Outliers were chosen by log_10_-transforming and removing values more than 1.5*IQR (inter-quartile range) beyond the 75^th^ percentile.

P-values were adjusted using the Bonferroni Correction method when performing multiple comparisons. Adjusted p-values greater than 1 are reported as 1. The standard alpha of 0.05 was used. To control for the possible effects of pseudoreplication we analyzed only the first interaction of each individual moth.

The number of individual bats frequenting the calibrated space at any given time varied from one to six. This resulted in some unavoidable pseudoreplication across individuals. This study covered a period of three years which should increase the probability of involving unique bats. We consider this an inevitable drawback of working with free-flying individuals.

## Supporting Information

S1 Table Breakdown of sample sizes used in each analysis. (PDF)

S2 Table Table 1 of reference [17]. (PDF)

S3 Table Table 2 of reference [26]. (PDF)
