# Supplementary material for: Acoustic Aposematism and Evasive Action in Select Chemically Defended Arctiine (Lepidoptera: Erebidae) Species: Nonchalant or Not?
Source: PLoS One. 2016 Apr 20;11(4):e0152981. doi: 10.1371/journal.pone.0152981 (PMC4838332; doi:10.1371/journal.pone.0152981)
Supplement: S1 Table — (PDF) [file pone.0152981.s001.pdf]

Data Subset	N	Figure	*P. roseicapitis* Tymbaled	*P. roseicapitis* Sham	*P. roseicapitis* Ablated	*C. martini* Tymbaled	*C. martini* Sham	*C. martini* Ablated
			**Breakdown By Species & Treatment**					
Filmed Interactions	167	2	50	12	25	33	30	17
Qualitative Evasive Response	87	7	26	15	17	17	5	7
			Tymbaled & Sham		Ablated	Tymbaled & Sham		Ablated
3-D Interaction Data	58	5 & 6	28		12	14		4
Moth Clicks Detected	20	Lines 128-135	14		0	6		0
Timing of Moth Clicks	15	3	9		N/A	6		N/A
Bat Calls Between Search Phases	19	4	6		6	5		2
