# Supplementary material for: Acoustic Aposematism and Evasive Action in Select Chemically Defended Arctiine (Lepidoptera: Erebidae) Species: Nonchalant or Not?
Source: PLoS One. 2016 Apr 20;11(4):e0152981. doi: 10.1371/journal.pone.0152981 (PMC4838332; doi:10.1371/journal.pone.0152981)
Supplement: S2 Table — (PDF) [file pone.0152981.s002.pdf]

Current Zoology 56 (3): 358–369, 2010

Anti-bat tiger moth sounds: Form and function

Aaron J. CORCORAN [1§], William E. CONNER [1*§], Jesse R. BARBER [2§]

[1] Department of Biology, Wake Forest University, Winston-Salem, NC 27106, USA

[2] Department of Fish, Wildlife and Conservation Biology, Colorado State University, Fort Collins, CO 80523, USA

Abstract The night sky is the venue of an ancient acoustic battle between echolocating bats and their insect prey. Many tiger moths (Lepidoptera: Arctiidae) answer the attack calls of bats with a barrage of high frequency clicks. Some moth species use these clicks for acoustic aposematism and mimicry, and others for sonar jamming, however, most of the work on these defensive functions has been done on individual moth species. We here analyze the diversity of structure in tiger moth sounds from 26 species collected at three locations in North and South America. A principal components analysis of the anti-bat tiger moth sounds reveals that they vary markedly along three axes: (1) frequency, (2) duty cycle (sound production per unit time) and frequency modulation, and (3) modulation cycle (clicks produced during flexion and relaxation of the sound producing tymbal) structure. Tiger moth species appear to cluster into two distinct groups: one with low duty cycle and few clicks per modulation cycle that supports an acoustic aposematism function, and a second with high duty cycle and many clicks per modulation cycle that is consistent with a sonar jamming function. This is the first evidence from a community-level analysis to support multiple functions for tiger moth sounds. We also provide evidence supporting an evolutionary history for the development of these strategies. Furthermore, cross-correlation and spectrogram correlation measurements failed to support a "phantom echo" mechanism underlying sonar jamming, and instead point towards echo interference [*Current Zoology* 56 (3): 358–369, 2010].

Key words Acoustic aposematism, Sonar jamming, Arctiidae, Cross-correlation, Autocorrelation

Many tiger moths produce high frequency clicks in response to the hunting calls of insectivorous bats. These sounds provide a survival advantage to their possessors (Dunning et al., 1992) but the sensory mechanisms by which anti-bat sounds work have spurred considerable debate (see Ratcliffe and Fullard, 2005). Throughout the discussion one or a few key species often have served as "representative". In reality there is considerable variation in the anti-bat sounds produced by tiger moths and it is this variation that is the focus herein. The questions that we address are: (1) how in parameter space do tiger moth sounds vary; (2) do the sounds fall into discrete categories; (3) what does the variation tell us about the functions of anti-bat sounds; and (4) how do the sounds vary across tiger moth taxa?

Tiger moths (Family: Arctiidae) produce sounds by activating bilateral thoracic blisters of cuticle called tymbal organs. The diversity of acoustic "displays" of arctiids have been documented by Blest (1964), Fenton and Roeder (1974), Fullard and Fenton (1977), and Barber and Conner (2006), but they have never been probed for consistent patterns within and across taxonomic groups. One key characteristic, the degree of frequency modulation, a characteristic that may be important to their function, has never been explored in a comparative way.

Tymbal organs (Fig. 1A) are thoracic plates modified by cuticular thinning and striation to produce a complex sound radiator (Blest et al., 1963; Fullard and Heller, 1990). During activation by underlying muscles, a wave of deformation expands from its dorsal origin ventrally along striations (Fenton and Roeder, 1974) producing a train of discrete clicks. After a brief pause, elastic recoil of the structure triggers a return wave across the same striations to produce a second burst of clicks (Fig. 1B). The number of clicks produced per full cycle is determined by the number of striations on the structure, a morphological trait, and the extent to which they are traversed, a behavioral trait. Species can vary in both traits and in the rate at which they activate the structure. The bilateral tymbals can also be activated synchronously or asynchronously (Fullard and Fenton, 1977). During a cycle the peak frequency of each click varies typically (but not always) from the high frequency to a

Received Dec. 09, 2009; accepted Feb. 17, 2010

§ All authors contributed equally and order of authorship was determined by lottery.

∗ Corresponding author. E-mail: conner@wfu.edu

© 2010 *Current Zoology*

Table 1 Tiger moth click parameters

Species	Sp. #	Site	N1	stdev kHz	swp	N2	MaxDC	N3	cdur	mhc	mc	isi	clicks	dB pe SPL	d kHz	-15 dB kHz	+15 dB kHz
<u>Subfamily Arctiinae</u>																	
Bertholdia femida	1	EC	4	7.9±0.7	0.9±0.2	5	30.9	5	0.26±0.04	7.3±0.3	22.1±0.9	7.4±0.7	7.2±0.8	88.4±0.8	53.9±7.5	47.6±6.7	64.1±3.3
Bertholdia trigona	2	AZ	6	11.4±1.8	3.9±0.6	6	43.8	6	0.28±0.07	12.2±3.2	29.5±5.9	5.3±1.0	21.5±5.4	80.7±2.1	48.7±8.7	21.9±10.9	83.1±5.6
Carales arizonensis	3	AZ	5	6.6±7.1	0.2±1.0	5	38.4	5	0.41±0.05	11.4±2.2	28.6±9.7	6.2±3.0	13.0±2.2	76.8±3.1	53.8±19.4	34.8±24.2	67.3±23.5
Cosmosoma stibasticta	4	EC	4	N/A	N/A	6	0.5	6	0.45±0.14	0.45±0.14	11.4±7.2	10.4±7.0	1.0±0.0	90.4±0.6	43.6±1.9	38.4±3.0	49.6±2.5
Ctenucha venosa	5	AZ	5	0.5±0.6	0.2±0.3	4	3.3	5	0.55±0.17	1.9±1.9	9.8±6.4	6.0±2.6	1.6±0.8	77.2±2.6	29.3±3.1	16.6±4.0	34.6±2.4
Cycnia tenera	6	NC	8	1.0±0.7	1.2±0.7	24	8.5	20	0.23±0.04	6.5±1.4	22.7±4.4	8.1±2.8	7.5±2.1	86.5±4.7	63.5±6.6	53.5±6.4	71.9±7.1
Eucereon aroa	7	EC	5	7.1±3.6	3.9±3.0	4	1.5	9	0.15±0.02	7.5±1.9	19.3±2.9	5.6±1.6	8.6±2.4	74.9±0.6	72.5±4.6	65.5±5.1	78.4±5.8
Eucereon decora	8	EC	5	5.4±3.7	0.0±0.4	2	9.9	10	0.19±0.03	14.0±2.8	36.3±6.8	7.1±1.5	20.3±5.1	85.7±2.4	54.5±3.8	43.5±6.7	63.6±3.9
Eucereon near *abdominale*	9	EC	4	0.7±0.6	0.2±0.1	3	1.4	4	0.51±0.02	10.6±2.1	56.5±14.0	13.6±4.2	6.8±3.5	89.6±0.2	30.5±1.9	25.1±2.5	34.0±2.0
Eucereon phaeproctum	10	EC	4	10.3±8.8	5.8±6.1	4	0.5	4	0.11±0.02	3.1±1.0	12.0±0.8	8.2±2.4	4.5±0.6	70.0±9.1	69.4±0.4	61.5±3.5	77.4±4.2
Eucereon tarona	11	EC	5	4.2±0.2	1.6±0.9	8	3.0	8	0.39±0.06	15.5±7.7	38.9±12.7	14.4±3.3	6.7±2.1	84.5±0.9	60.5±6.2	53.5±4.6	70.8±6.1
Euchaetes egle	12	NC	5	2.3±1.9	0.3±0.4	4	3.1	5	0.17±0.02	12.6±3.9	40.5±4.7	14.4±2.7	6.4±1.8	78.3±2.8	66.3±4.4	47.0±7.1	80.1±7.1
Euchaetes antica	13	AZ	6	7.0±5.0	1.2±1.0	1	4.0	6	0.17±0.03	6.0±1.9	19.6±4.7	6.5±3.1	8.5±4.1	78.6±3.0	62.5±4.6	24.2±7.6	78.1±7.4
Gymnelia sp. 1	14	EC	5	0.3±0.6	0.2±0.4	13	3.8	13	0.41±0.13	1.8±3.4	11.8±8.1	9.2±7.0	1.8±1.9	67.6±4.1	40.8±2.3	34.6±4.7	46.2±5.3
Halysidota near *cirphis*	15	EC	4	5.6±2.2	2.4±1.8	5	3.4	5	0.18±0.02	10.3±2.2	33.9±6.1	9.3±3.7	17.4±6.5	85.5±2.9	51.2±3.6	39.9±9.2	59.1±4.8
Hemihyalea edwardsii	16	AZ	6	0.6±0.4	0.3±0.2	N/A	N/A	6	0.33±0.12	6.1±6.9	29.5±17.7	19.2±5.7	2.4±1.2	84.5±4.6	82.2±17.3	67.2±9.8	101.9±18.3
Hemihyalea near *alba*	17	EC	5	0.5±0.6	0.0±0.2	N/A	N/A	6	0.28±0.07	9.4±2.8	30.3±7.0	13.1±2.9	7.2±1.8	90.1±0.1	27.7±2.6	23.5±1.6	32.6±2.2
Idalus near *veneta*	18	EC	5	10.7±2.8	6.9±7.4	4	35.8	4	0.38±0.01	6.8±1.6	21.1±2.7	8.9±1.5	5.6±0.9	89.0±3.2	44.7±5.0	39.3±2.6	50.3±6.6
Ischnocampa sp. 2	19	EC	4	11.1±2.2	0.1±0.2	2	50.7	10	0.27±0.04	5.8±1.2	16.0±4.6	4.1±1.0	8.9±1.1	86.0±2.3	54.5±5.9	46.0±8.0	63.1±7.8
Melese near *drucei*	20	EC	5	7.6±3.3	2.4±1.0	13	23.9	13	0.19±0.04	9.6±1.1	24.9±2.5	5.0±1.1	11.6±1.7	76.7±0.7	77.8±3.0	64.3±6.5	90.1±3.7
Pygarctia roseicapitis	21	AZ	5	2.8±1.4	-0.80.9	4	6.1	5	0.20±0.03	10.0±2.2	38.4±8.2	18.7±5.1	3.5±0.4	76.9±4.8	54.0±8.4	24.1±6.2	75.2±21.6
Virbia fragilis	22	AZ	8	6.5±5.5	2.1±2.7	5	4.5	8	0.12±0.03	6.1±3.5	22.1±10.5	9.4±7.4	6.0±2.4	69.1±6.0	60.6±11.3	33.3±11.7	109.5±9.7
<u>Subfamily Lithosiinae</u>																	
Amplicincia near *mixta*	23	EC	5	3.4±1.7	1.6±1.6	2	0.3	8	0.11±0.02	3.7±1.1	12.9±2.0	5.1±0.8	6.4±3.0	76.6±0.1	72.2±3.8	63.9±6.3	79.4±6.6
Cisthene martini	24	AZ	7	5.2±5.8	0.6±1.5	4	5.7	7	0.25±0.07	4.1±1.6	16.7±1.8	8.9±2.2	4.9±1.4	74.6±7.7	60.9±7.9	34.5±11.2	73.3±9.3
Cisthene tenuifaschia	25	AZ	6	4.5±4.2	0.8±1.5	6	11.6	6	0.33±0.05	3.9±1.5	14.3±0.8	6.3±2.3	4.8±2.2	72.0±6.4	58.4±5.5	35.2±13.8	68.8±7.0
Crambidia sp.	26	EC	4	3.8±2.7	1.5±2.0	N/A	N/A	4	0.15±0.03	6.1±0.8	15.5±1.6	5.6±1.9	10.0±1.8	78.3±0.7	70.2±4.2	61.3±6.1	76.3±1.5

All temporal values are listed in ms. Abbreviations: Sp. #, species number; Site, collection site; AZ, Arizona; EC, Ecuador; NC, North Carolina; N1, sample size for frequency modulation measurements; stdev kHz, standard deviation of click frequency in kHz; sweep, click sweep rate in kHz/ms; N2, sample size for duty cycle measurements; MaxDC, maximum duty cycle over 100 ms interval; N3, sample size for modulation cycle parameter measurements; cdur, click duration; mhc, active half modulation cycle duration; mc, modulation cycle duration; isi, inter-cycle silent interval; clicks, number of clicks in active half modulation cycle; dB pe SPL, peak equivalent sound pressure level in decibels re. 20 μPa; d Khz, dominant frequency in kHz; -15 dB kHz, frequency -15 dB below d kHz; +15 dB kHz, frequency -15 dB above d kHz. Values are mean ± standard deviation.
