# Supplementary material for: Acoustic Aposematism and Evasive Action in Select Chemically Defended Arctiine (Lepidoptera: Erebidae) Species: Nonchalant or Not?
Source: PLoS One. 2016 Apr 20;11(4):e0152981. doi: 10.1371/journal.pone.0152981 (PMC4838332; doi:10.1371/journal.pone.0152981)
Supplement: S3 Table — (PDF) [file pone.0152981.s003.pdf]

DARTMOUTH COLLEGE LIBRARY

Canadian Journal of Zoology

Published by

THE NATIONAL RESEARCH COUNCIL OF CANADA

Journal canadien de zoologie

Publié par

LE CONSEIL NATIONAL DE RECHERCHES DU CANADA

Volume 55 Number 8 August 1977

Volume 55 numéro 8 août 1977

Acoustic and behavioural analyses of the sounds produced by some species of Nearctic Arctiidae (Lepidoptera)

J. H. FULLARD AND M. B. FENTON

Department of Biology, Carleton University, Ottawa, Ont., Canada K1S 5B6

Received February 7, 1977

FULLARD, J. H., and M. B. FENTON. 1977. Acoustic and behavioural analyses of the sounds produced by some species of Nearctic Arctiidae (Lepidoptera). Can. J. Zool. **55**: 1213–1224.

Seventeen of the 24 species of Nearctic Arctiidae which we examined produced sounds under a variety of tactile and acoustic stimuli. Seven of 14 sound-producing species tested emitted their sounds in response to the hunting cries of insectivorous bats or simulations thereof. The sounds were generated by the buckling action of the microtymbal band (=striated band) on the surface of the modified thoracic metepisternum (=tymbal). Sound production is not dependent on the presence of microtymbals as four species of *Callarctia* possessed nonstriated, functional tymbals. In contrast, other species possessed striated, apparently nonfunctional tymbals.

The sounds of the arctiids we studied were predominantly ultrasonic (>20 kHz) and relatively faint (<85 dB at 2 cm) compared with other insect sounds.

The acoustic parameters of the arctiids surveyed revealed extremely high levels of variability in the sounds. Smaller species generally emit higher pulse repetition rates and more intense signals than larger ones. The behavioural contexts in which the sounds are produced and their relation to arctiid defense are discussed.

FULLARD, J. H., et M. B. FENTON. 1977. Acoustic and behavioural analyses of the sounds produced by some species of Nearctic Arctiidae (Lepidoptera). Can. J. Zool. **55**: 1213–1224.

Dix-sept des 24 espèces d'arctiidés néarctiques examinés produisent des sons sous l'action de divers stimuli tactiles ou auditifs. De 14 espèces émettrices de sons, 7 produisent leurs sons en réaction aux cris de chasse de chauves-souris insectivores ou en réaction à une simulation de ces cris. Les sons viennent du repliement de la bande striée sur la surface du métépisterne thoracique modifiée (timbale). La production de son ne dépend pas de la présence des stries, puisque quatre espèces de Collarctia possèdent des timbales fonctionnelles non striées. Par contre, d'autres espèces possèdent des timbales striées qui ne semblent pas être fonctionnelles.

Les sons des arctiidés observés sont surtout des ultra-sons (>20 kHz) et sont relativement faibles (<85 db à 2 cm) par rapport aux sons d'autres insectes.

Les paramètres auditifs des espèces étudiées mettent en évidence le très haut degré de variabilité des sons. Les espèces plus petites émettent généralement des sons de plus haute intensité à une fréquence de répétition plus élevée que les grosses espèces. On discute des comportements qui entourent l'émission de ces sons et on tente de les relier au comportement de défense des arctiidés.

[Traduit par le journal]

Introduction

Arctiid moths are generally regarded as distasteful, visually aposematic insects (Frazer and Rothschild 1960; Rothschild 1961, 1963, 1965). Sounds produced by arctiids were first reported by Laboulbène (1864) and subsequently described in detail for a variety of species (Blest *et al.* 1963; Blest 1964). They concluded that the sounds of the moth *Melese laodamia* were generated by the buckling action of the 'striated band' (Forbes and Franclemont 1957) on the thoracic metepisternum (= tymbal)

Can. J. Zool. Downloaded from www.nrcresearchpress.com by McMaster University on 11/19/14
For personal use only.

Can. J. Zool. Downloaded from www.nrcresearchpress.com by McMaster University on 11/19/14
For personal use only.

TABLE 2. Acoustic parameters of arctiid emissions

Species	A/S[a]	MHC[b]	MC[c]	ICSI[d]	P/S[e]	P/MHC[f]	INT[g]	DFREQ[h]
Cycnia tenera	A	5.3±1.0[i] (10)	18.6±2.7 (10)	6.7±1.8 (10)	1497.1±269.4 (10)	7.7±2.0 (10)	68.5±4.0 (8)	53.0±16.4 (10)
Euchaetias egle	A	11.0±3.6 (5)	34.8±9.3 (5)	21.4±20.6 (5)	658.6±319.1 (10)	6.4±2.6 (10)	73.0±4.2 (2)	54.0±13.4 (10)
E. oregonensis	A	4.1±1.2 (3)	17.8±3.8 (3)	5.2±0.5 (3)	1708.8±142.5 (3)	6.9±2.5 (3)	70.1 (1)	63.0±10.6 (3)
Halysidota tessellaris	A	6.1±1.9 (10)	23.1±4.6 (10)	15.1±3.9 (10)	718.2±235.6 (10)	3.8±1.9 (10)	66.5±7.8 (2)	72.2±4.6 (10)
Pyrrharctia isabella	S	7.9±1.7 (7)	31.9±10.5 (7)	15.0±10.2 (7)	1016.6±342.7 (7)	7.2±2.3 (7)	54.0 (1)	42.6±11.0 (7)
Phragmatobia rubicosa	A	4.5 (1)	13.1 (1)	7.0 (1)	649.4 (1)	2.8 (1)	67.5 (1)	40.0 (1)
Callarctia anna	A	—[j] (2)	—[k] (2)	—[k] (2)	20.2 (1)	1.0 (1)	56.5 (1)	62.0 (1)
C. arge	A	—[j] (1)	—[k] (1)	—[k] (1)	13.7 (1)	1.0 (1)	69.5 (1)	35.0 (1)
C. virgo	A	—[j] (2)	—[k] (2)	—[k] (2)	86.5 (1)	1.0 (1)	66.5 (1)	32.5±3.5 (2)
C. virguncula	A	—[j] (2)	—[k] (2)	—[k] (2)	16.8±16.9 (2)	1.0±0.0 (2)	—[l]	30.0 (1)
Haploa confusa	A	5.5±2.6 (3)	45.3±21.9 (3)	37.0±27.5 (3)	703.6±89.9 (3)	3.8±2.4 (3)	67.7±1.1 (2)	51.3±14.4 (3)
H. contigua	A	7.8 (1)	51.5 (1)	32.7 (1)	618.7 (1)	4.4 (1)	63.5 (1)	35.0 (1)
Crambidia pallida	A	3.1±0.5 (2)	13.1±1.3 (2)	6.2±1.7 (2)	1581.8±176.6 (2)	4.3±1.0 (2)	67.5 (1)	39.5±0.7 (2)
Hypoprepia fucosa	A	6.4±1.1 (11)	22.5±3.9 (11)	9.9±2.0 (11)	972.9±227.1 (11)	6.1±1.6 (11)	77.9±6.3 (11)	49.4±4.3 (11)
Lycomorpha pholus	A	8.7 (1)	23.5 (1)	8.0 (1)	916.4 (1)	8.0 (1)	82.5 (1)	45.0 (1)

NOTE: Values are from five complete modulation cycles from each individual.
[a]Indicates operating sequence of tymbals. A, alternant; S, synchronous.
[b]Duration of active modulation half cycle (milliseconds).
[c]Duration of modulation cycle (milliseconds).
[d]Duration on intracycle silent interval (milliseconds).
[e]Pulses per second.
[f]Pulses per active modulation half cycle.
[g]Intensity measured at 2 cm (linear setting) in dB (re 2×10^{-5} N/cm^2).
[h]Dominant frequency (maximally intense spectral peaks).
[i]Mean ± SD; figures in parentheses indicate sample size.
[j]MHC consists of only one pulse.
[k]Parameter too variable for precise measurement.
[l]Parameter not measured.
